# 3D Bioprinted Head and Neck Squamous Cell Carcinoma (HNSCC) Model Using Tunicate Derived Nanocellulose (NC) Bioink

**DOI:** 10.1002/adhm.202403114

**Published:** 2025-01-13

**Authors:** Alexya Azhakesan, Johann Kern, Ankit Mishra, Christine Selhuber‐Unkel, Annette Affolter, Paul Gatenholm, Nicole Rotter, Karen Bieback

**Affiliations:** ^1^ Medical Faculty of Mannheim University of Heidelberg Department of Otorhinolarynlogy Head and Neck Surgery 68167 Mannheim Germany; ^2^ Institute for Molecular Systems Engineering and Advanced Materials (IMSEAM) Heidelberg University 69120 Heidelberg Germany; ^3^ 3D Bioprinting Centre Department of Chemistry and Chemical Engineering Chalmers University of Technology Gothenburg 41296 Sweden; ^4^ Institute of Transfusion Medicine and Immunology Medical Faculty Mannheim Heidelberg University German Red Cross Blood Donor Service Baden‐Württemberg – Hessen 68167 Mannheim Germany

**Keywords:** 3D Bio‐printing, 3D tumor model, bioink, head and neck squamous cell carcinoma, nanocellulose

## Abstract

Head and neck squamous cell carcinoma (HNSCC) are invasive solid tumors accounting for high mortality. To improve the clinical outcome, a better understanding of the tumor and its microenvironment (TME) is crucial. Three ‐dimensional (3D) bioprinting is emerging as a powerful tool for recreating the TME in vitro. To establish long‐term HNSCC bioprinted constructs for personalized drug‐testing, this proof‐of‐principle study aims to compare two different innovative tunicate‐derived nanocellulose (NC) hydrogels against the widely used semi‐synthetic gelatin methacryloyl (GelMA). Cell lines of different tumor origin sites are printed in TEMPO and Carboxy‐NC, and GelMA in alginate (GelMAA). Both NC hydrogels show higher bioprintability than GelMAA. Carboxy‐NC supported long‐term HNSCC survival, proliferation, and maintenance of epithelial phenotype in 3D bioprinted constructs similar to GelMAA. The hydrogel microstructure revealed differences in pore size. Importantly, the established HNSCC bioprinted model allowed the testing of radiochemotherapy (RCT) both in cell lines and patient‐derived cultures. Compared to a spheroid model, the cytotoxic effects are less, better reflecting the response in patients. The proof‐of‐principle findings indicate that Carboxy‐NC is a viable alternative to gelatin‐based bioink with improved bioprintability allowing personalized drug‐testing. By adding other cell‐types of the TME, this model can be advanced to a heterotypic one.

## Graphical abstract

A 3D bioprinted HNSCC model for use in anti‐cancer drug testing is established in proof‐of‐concept. The innovative tunicate‐derived nanocellulose hydrogel proved to be a viable alternative to gelatin‐based hydrogel, offering improved bioprintability for HNSCC model establishment. The bioprinted construct allows better prediction of radiochemotherapy response than the alternatively used 3D spheroid models.

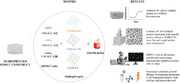

## 1 Introduction

Head and neck squamous cell carcinoma (HNSCC) are invasive solid tumors with 5‐year survival rates of 45–60%.^[^ ^1^ ^]^ Conventional treatment strategies include‐ surgical resection, chemo‐ and/ or radiotherapy, and immune‐therapy, mostly in combination.^[^ ^1^, ^2^ ^]^ However, treatment failures are often observed, most likely related to individual‐to‐individual tumor variability. Thus, there is a need for better and optimized therapeutic strategies that take these individualities into account. Especially, long‐term models are needed for high‐throughput drug testing/ toxicology studies that could be used in personalized medicine.^[^ ^2^, ^3^, ^4^, ^5^ ^]^ With a specific focus on recapitulating the HNSCC tumor microenvironment/niche (TME), three‐dimensional (3D) models have been introduced including‐ spheroids, organoids, 3D bioprinted models, and organ‐on‐a‐chip (Table S1, Supporting Information).^[^ ^3^, ^4^, ^5^, ^6^ ^]^ By using patient‐derived cells, they offer the unique potential to test drugs in a personalized approach.

The ultimate aim of the research study is to develop a 3D printed HNSCC model.^[^ ^6^ ^]^ Specifically, with 3D bioprinting technology we can design and print structures (with living cells and biomaterials) in a custom manner with a defined geometry that replicates the tumor niche.^[^ ^4^, ^6^ ^]^ Therefore, unlike 3D organoids and spheroids, a bioprinted 3D system offers a well‐defined and reproducible milieu for instance, for time efficient high‐throughput drug screening, toxicological studies, gene therapy, and personalized medicine. There are different methodologies to fabricate a 3D bioprinted model such as‐ extrusion‐based, laser‐based, stereo lithography, light‐based, and droplet‐based bioprinting processes. In this project, we opted for extrusion‐based bioprinting.^[^ ^4^, ^5^, ^6^ ^]^ Briefly, extrusion‐based bioprinting uses a pneumatic/ screw/ piston‐based extrusion pump to extrude continuous filaments of the bioink in a layer‐by‐layer fashion.^[^ ^4^, ^5^, ^6^ ^]^ Until now, very few HNSCC 3D bioprinted models have been published.^[^ ^7^ ^]^

Based on our group's research work on the usability of tunicate‐nanocellulose (NC) in tissue engineering, which has proven biocompatibility and high shape fidelity, we chose to test it as a bioink for bioprinting a HNSCC model.^[^ ^8^ ^]^ We focus on tunicate NC, because we finally aim to develop a heterotypic TME model that incorporates various immune cell subsets. Therefore, it is necessary to choose an immunologically inert hydrogel. Tunicate‐derived NC has been proven its efficacy already in various tissue engineering approaches, where it induced no inflammatory response and specifically no macrophage activation by endotoxins.^[^ ^9^, ^10^, ^11^, ^12^ ^]^ Tunicate NC contains cellulose without hemicellulose and lignin.^[^ ^9^ ^]^ Further, it offers specific biochemical and mechanical advantages, i.e.,‐ low toxicity, high surface area, surface tunable chemistry, good mechanical strength with high Young's and storage modulus, and high‐water retention capacity with high biocompatibility and low‐to no cytotoxicity inducing no to low immune response.^[^ ^9^, ^13^, ^14^ ^]^ The NC source and extraction method defines its physiochemical and mechanical properties.^[^ ^13^, ^14^, ^15^, ^16^ ^]^ To enhance NC‐biocompatibility, NC can be surface functionalized according to the desired applications‐ acetylation, etherification, sulfonation, oxidation, carboxymethylation, and phosphorylation.^[^ ^10^ ^]^ 2,2,6,6 Tetramethylpiperidinyloxy (TEMPO)‐oxidated and carboxy‐methylated tunicate NC were chosen for this study as they possess negative surface charges favoring long‐term cultures.^[^ ^10^, ^17^ ^]^

The widely used gelatin methacrylate (GelMA) was used for comparison. It has been reported to have improved biomechanical properties and shape fidelity in conjugation with polymers such as alginate, dextran, methacrylic anhydride, polyethylene glycol diacrylate (PEGDA), and polylactide (PLA).^[^ ^18^, ^19^ ^]^ But, GelMA has demonstrated to cause immune modulation by eliciting an inflammatory cytokine response.^[^ ^20^ ^]^ This reaction might disrupt the interactions that take place between immune cells, the extra‐cellular matrix and tumor cells within a heterotypic 3D bioprinted tumor model. Consequently, we opted for NC hydrogel based on this significant advantage over GelMA.

**Figure 1:**
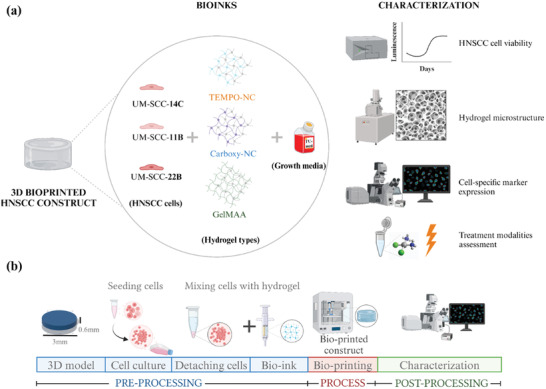
Schematic representation of the 3D bioprinted head and neck squamous cell carcinoma (HNSCC) model‐ a) overall model establishment; b) step‐by‐step process involved in fabricating the 3D bioprinted model. The 3D biofabrication process can be divided into pre‐processing, process, and post‐processing steps. Initially, a 3D structure (3D cylinder‐ 3 × 0.6 mm dimensions) was designed using a 3D designing software. Then, bioink formulations were prepared by mixing different HNSCC cell types (UM‐SCC‐14C, 11B, and 22B) and the respective hydrogel mixtures (NC‐& gelatin‐based hydrogels in alginate). The prepared bioink was loaded onto the bioprinting cartridges and printed in the form of a 3D cylinder. Finally, the bioprinted constructs were characterized for their viability, cell proliferation, cell‐specific biomarker expression, and differences in cell‐free hydrogel microstructure. (TEMPO‐NC: TEMPO‐oxidized tunicate derived nanocellulose; Carboxy‐NC: Carboxymethylated tunicate derived nanocellulose; GelMAA: Gelatin methacrylate with alginate) (Illustration created in Biorender).

The specific aim of this study is to achieve a 3D bioprinted HNSCC model as proof‐of‐concept to test whether HNSCC can be bioprinted, survive in the long‐term (at least 21 days allowing for instance for radiation experiments and clonal selection studies) and maintain their epithelial phenotype.^[^ ^21^ ^]^ First, we compared two different NC‐based bioinks, namely TEMPO‐oxidized tunicate derived NC and carboxy‐methylated tunicate‐derived NC with the broadly used GelMA. Second, we compared three HNSCC cell lines of different tumor sites. We investigated the survival of HNSCC cells in the different bioink‐bioprinted constructs by monitoring their viability, cellular distribution, proliferation and cell specific biomarker expression. Third, we determined the microstructure of the three hydrogels. Finally, we assessed the susceptibility of the bioconstructs to radiochemotherapy (RCT)‐ cell lines and patient‐derived HNSCCs (**Figure** **1**). To advance the model further to a patient‐specific multicellular model as drug testing system, after recreating the TME, immune cell subsets will be added.

## 2 Results and Discussion

### 2.1 Investigation of Bioprinting Parameters

Toward achieving a bioprinted construct retaining its shape fidelity over a 21‐day long culture period, we optimized printing parameters such as printing temperature & speed, infill density, number of layers, printing pattern, gelation time & temperature, nozzle diameter, crosslinking method, etc., as summarized in **Table** **1**.^[^ ^22^, ^23^, ^24^ ^]^ Both tunicate‐ NC bioinks showed experimental ease of use, good bioprintablility and long‐term stability.

**Table 1:** Optimized bioprinting parameters for fabricating 3D HNSCC model.

Bioprinting parameters	Ranges tested	Optimized Value
NC‐ gelation temperature (°C)	20–26 °C	26 °C
GelMAA‐ gelation temperature (°C)		26 °C (GelMAA‐ in a temperature controlled printhead)^a)^
Printing temperature (°C)		26 °C (R.T)
Print‐head temperature (°C)		26 °C (R.T)
Print‐bed temperature (°C)		10 °C (GelMAA bioink)^a)^
Infill density (%)	60‐90	75–85
Printing speed (mm s^−1^)	2–6	3–4
Printing pressure	5–20	6–12
Number of layers	1–2	1
Printing pattern	Concentric, grid & honeycomb	Concentric
Crosslinking method		Chemical‐ CaCl_2_
Needle/ Nozzle inner diameter (µm)	0.025–0.041	0.041
Growth media volume (µL)	50–200	200

^a)^ (as per the manufacturer's protocol)

To closely replicate the native tumor situation, a 3D model with cells interacting with each other is essential. Cell density/mL bioink is known to play a crucial role in affecting viability and shape fidelity.^[^ ^22^, ^23^, ^24^, ^25^ ^]^ Hence, defining a suitable cell density, which at same time balanced with the bioink printability was the first major challenge addressed in this study.

**Figure 2:**
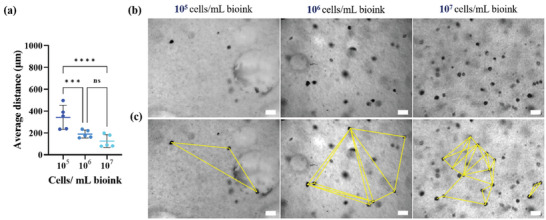
Optimization of cell density/mL bioink for bioink preparation. a) The average cell‐to‐cell distance between the neighboring cells were measured and plotted (mean with standard deviation (SD)). b,c) The bright field microscopic images of HSNCC cells in bioconstructs with c) indicating the cell‐cell distance measurement map. (*n* = 1 experiment with 5 technical replicates) (Scale: 50 µm) (2‐way ANOVA analysis; ns‐ non‐significant; ^***^ *p* <  0.0005 and ^****^ *p* < 0.0001).

To bioprint HNSCC cells with a close cell‐to‐cell distance, we tested different cell densities/ mL bioink (1 × 10^5^, 1 × 10^6^, 1 × 10^7^ UM‐SCC‐11B cells mL^−1^ of bioink) crosslinked with 50 mm calcium chloride (CaCl_2)_. After 24 h, the bioprinted HNSCC constructs were imaged, and their cell‐to‐cell distance within the bioconstructs was measured in one focal plane (**Figure** **2**). While there was a significant decrease in average cell‐to‐cell distance between 1 × 10^5^ and 1 × 10^6^ cells mL^−1^ bioink, we did not observe a significant difference between 1 × 10^6^ and 10^7^ cells mL^−1^ bioink. Bioprinting with 1 × 10^7^ cells mL^−1^ of bioink, however, sometimes resulted in clogging of the nozzle. This affected printability and shape fidelity of the bioprinted constructs. Accordingly, we chose 5 × 10^6^ cells/mL^−1^ bioink. This cell number did not lead to impaired printability and allowed to bioprint the required number of replicates needed for further investigations to monitor data reproducibility.

### 2.2 Bioprinting Lowers but Maintains HNSCC‐Cell Type Specific Metabolic Activity/Viability

Having optimized the cell density/mL bioink, we assessed cell viability by measuring metabolic activity (adenosine triphosphate (ATP) content within the cells). We first compared data from different cell numbers in both conventional 2D culture to the 3D bioprinted HNSCC construct to document a linear relationship between the cell number and the luminescence signal. To represent the heterogeneity and various localizations of HNSCC tumors, two different HSNCC cell lines‐ UM‐SCC‐14C, and 11B derived from oral cavity, and larynx respectively, and a lymph node metastatic cell line‐ UM‐SCC‐22B derived from hypopharynx, were chosen.^[^ ^26^ ^]^ The chosen cells were seeded at‐ 5000, 10 000, 15 000, and 20 000 cells well^−1^ in a 96‐well plate. Furthermore, we 3D bioprinted them with the following cell numbers‐ 1 × 10^6^, 2 × 10^6^, 3 × 10^6^, 4 × 10^6^, and 5 × 10^6^ cells mL^−1^ bioink (corresponding to 4200, 8400, 12 600, 16 800, and 21 000 cells/ bioconstruct, respectively). The constructs printed in the form of 3D cylinders were crosslinked with 50 mm CaCl_2_. After 24 h, viability was assessed for both 2D and 3D HNSCC samples.

**Figure 3:**
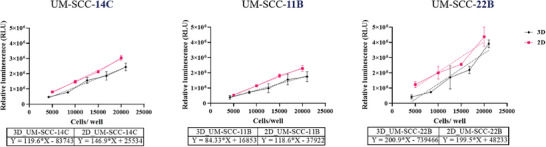
Bioprinting lowers but maintains HNSCC‐cell type specific metabolic activity/viability. The viability of HNSCC cells is cell‐line specific and follows a similar pattern in 2D and 3D. The relative luminescence values (RLU) were plotted against cells/ well and fitted in a simple linear regression model. UM‐SCC‐14C (3D: *y* = 119.6*x – 83 743; 2D: *y* = 146.9*x + 25 534), UM‐SCC‐11B cells (3D: *y* = 84.33*x + 16 853; 2D: *y* = 118.6*x – 37 922), UM‐SCC‐22B (3D: *Y* = 200.9*X – 739 466; 2D: *Y* = 199.5*X + 48 233) (*n* = 12 samples per condition) (2‐way ANOVA analysis).

Plotting the relative luminescence unit (RLU) against the cell number/well, we observed a linear relationship between the RLU and the cells/well which was consistent both in 2D and 3D HNSCC models for every HNSCC cell line (**Figure** **3**). Comparing 3D and 2D bioprinted HNSCC models we observed a 30.8% and 33.9% reduced luminescence, respectively in UM‐SCC‐14C and 11B cells. For UM‐SCC‐22B cells, the luminescence signal was 41.8% lower in 3D than in 2D. The cell‐free constructs gave no detectable background signal. Hence, we argue that the 3D hydrogel construct may have reduced the chemiluminescence signal by scattering or signal absorption/ refraction.^[^ ^27^ ^]^ Alternatively, the reduced signal may indicate distress of the cells, possibly due to the shear stress experienced during bioprinting.^[^ ^24^, ^25^ ^]^

The luminescence intensity emitted by different HNSCC cell lines differed significantly, both in 2D and 3D. Since the luminescence directly correlates to the metabolic activity, we can infer that every HNSCC cell type produces different levels of ATP. In 2D, both UM‐SCC‐14C and 11B cells grew at a much faster pace with a 3x doubling time of than the UM‐SCC‐22B cells. The different metabolic activities may relate to the tissue origin and grade of the tumor from which the cell lines have been generated. Higher ATP concentration has been observed in TME than in a normal/ healthy extracellular environment, contributing to tumor metastasis.^[^ ^28^ ^]^ Meanwhile, lower ATP levels have been taken as an indicator for tumor proliferation and immune suppression.^[^ ^28^ ^]^ UM‐SCC‐22B cells were derived from lymph node metastasis and UM‐SCC‐11B cells from larynx carcinoma. Possibly the high ATP level could indicate tumor metastasis, whereas the low ATP level may correspond to tumor proliferation. However, to prove this assumption, more tissue samples need to be investigated.

### 2.3 Carboxy‐NC Supports Long‐Term HNSCC Survival in 3D Bioprinted Constructs Similar to GelMAA Depending Upon the HNSCC Line Used and the Crosslinker Concentration

**Figure 4:**
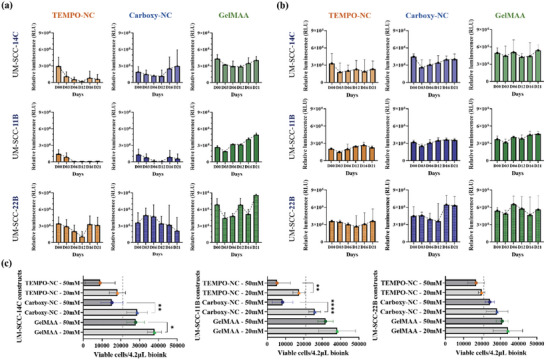
Carboxy‐NC supports long‐term HNSCC survival in 3D bioprinted constructs similar to GelMAA depending upon the cell line used. Viability of different HNSCC cell lines over 21 days in different bioinks crosslinked with a) 50 mm CaCl_2_ and b) 20 mm CaCl_2._ c) Comparison of HNSCC viability in different bioinks at the end of 21 days according to the two different crosslinker concentrations. The line indicates the initial bioprinted cell number (= 21 000 cells/ / 4.2 µL bioink). N‐3 independent experiments; *n* = 3 samples per condition ^*^ *p* < 0.05, ^**^ *p* < 0.005, ^***^ *p* < 0.0005, ^****^ *p* < 0.0001 (2‐way ANOVA analysis).

As a next step, we cultured the bioprinted constructs for a period of 21 days and monitored their viability/metabolic activity on day (D) 00, 03, 06, 12, 16, and 21, comparing a) bioinks, b) cell lines and c) crosslinker concentrations (**Figure** **4**). To compare the HNSCC cells in different bioinks, we bioprinted three HNSCC cell lines in TEMPO‐NC, Carboxy‐NC, and GelMAA bioinks (Figure 4a). GelMAA bioink required precisely defined gelation temperature, printhead temperature, printing in pre‐cooled plates, higher printing pressure and speed. Overall, GelMAA bioprinting reproducibility was lower than tunicate‐NC.

Regarding UM‐SCC‐14C, we observed a rapid decline in the viability in TEMPO‐NC. On D16, a small rise was seen slightly dropping again on D21. The curves in Carboxy‐NC indicated a slight loss in viability until D12, afterward cells recovered showing an increase in viability until D21 indicative of proliferation. This profile was similar in GelMAA. However, in GelMAA, the initial RLU was much higher than in TEMPO‐ NC and Carboxy‐NC (4 × 10^6^, 3 × 10^6^, and 1.8 × 10^6^ respectively for D00). UM‐SCC‐11B showed a similar decline in cell viability in TEMPO‐NC, yet the cells did not recover over the 21‐day period. In Carboxy‐NC, cells slightly recovered on D16 and D21. In GelMAA, we observed an initial drop in viability on D03. Thereafter, the cells displayed a linear increase in viability/metabolic activity until D21. Again, the D00 RLU values in GelMAA were ≈2‐fold higher in UM‐SCC‐22B than in TEMPO‐NC and Carboxy‐NC bioconstructs. The viability of UM‐SCC‐22B cells linearly decreased until D12 but it recovered on D16 and D21 to the initial viability values. In Carboxy‐NC, an entirely contrary profile was observed. Here, cells showed an increased viability rather than a decrease on D03 and D06. Thereafter, the viability gradually decreased. In GelMAA, a biphasic pattern was observed. An initial drop in viability on D03 and D06, a rise in viability back to the input value (21 000 cells/ bioconstruct) on D08, a drop on D16, and again an increase to values higher than the input viability on D21.

The large differences in viability of UM‐SCC‐11B and 14C in TEMPO‐NC and Carboxy‐NC compared to GelMAA need to be explained. We hypothesize that cells suffered from bioprinting or from the crosslinker concentration.^[^ ^25^, ^27^, ^29^ ^]^ Also, we observed vacuole formation in the 3D bioprinted UM‐SCC‐11B constructs (Figure S1, Supporting Information). According to the literature, a high concentration of calcium in a cell could cause cell membrane damage leading to disturbed state of cell electrolytes, thus contributing to vacuolization and gradually, cell death.^[^ ^27^ ^]^ Accordingly, we lowered the concentration of the ionic crosslinker CaCl_2_ from 50  to 20 mm.

Reducing the chemical crosslinker concentration, we observed a substantial increase in the viability (Figure 4). This was especially obvious for the UM‐SCC‐11B bioconstructs. Further, the HNSCC bioconstructs appeared to recover much earlier from printing shear stress as evidenced by an increase in viability already on D03. After D03 they seemed to proliferate until D12, later reaching a state of stagnant growth by D21 for UM‐SCC‐14C and 11B NC bioconstructs. In UM‐SCC‐22B NC bioconstructs, we observed a gradual decline in the luminescence until D12, when cells started proliferating until D21. There was no significant difference between Carboxy‐NC and GelMAA bioinks. The TEMPO‐NC bioconstructs, when crosslinked with 20 mm CaCl_2_, supported HNSCC cell survival significantly better than those crosslinked with 50 mm CaCl_2_.^[^ ^27^, ^29^ ^]^ With 20 mm CaCl_2_, the viability profiles of UM‐SCC‐14C and 11B were very similar in all three hydrogels (Figure 4b). They showed an initial drop in viability on D03, and then recovered to approximately input values on D21. Interestingly, the differences in input viability between GelMAA and NC‐hydrogels, were not as prominent (for example in UM‐SCC‐14C: 4.5 × 10^6^, 4 × 10^6^, and 3 × 10^6^ for GelMAA, Carboxy‐ NC and TEMPO‐NC respectively). In UM‐SCC‐22B, on the contrary, we observed a low gradual decrease in cell viability until D12 and an increase on D16 and D21 in NC‐hydrogels. In GelMAA, cell viability fluctuated around the initial input value.

These data clearly demonstrate that the different HNSCC cell lines behave differently in different bioinks. Furthermore, cell survival can be significantly increased by lowering the crosslinker concentration (Figure 4c). Only Carboxy‐NC crosslinked with 20 mm CaCl_2_ supported an increase in viability for 21‐days, indicative of proliferation. GelMAA promoted long‐term HNSCC cell survival and proliferation consistently and largely irrespective of the crosslinker concentration (Figure 4c).

In conclusion, we favor Carboxy‐NC bioink which behaves similar to GelMAA but provided much better bioprintability.

**Figure 5:**
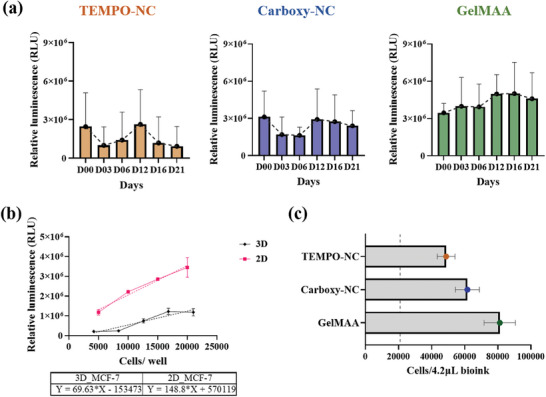
Carboxy‐NC supports long‐term MCF‐7 cell survival in 3D bioprinted constructs similar to GelMAA depending upon the cell line used. Viability of MCF‐7 cells over 21 days in different bioinks crosslinked with 20 mm CaCl_2_ a,b) the viability of MCF‐7 cells follows a similar pattern in 2D and 3D (3D: *y* = 69.63*x – 153 473; 2D: *y* = 148.8*x + 570 119). c) Comparison of HNSCC viability in different bioinks at the end of 21 days. The line indicates the initial bioprinted cell number (= 21 000 cells/ / 4.2 µL bioink). N‐3; *n* = 3 samples per condition ^*^ *p* < 0.05, ^**^ *p* < 0.005, ^***^ *p* < 0.0005, ^****^ *p* < 0.0001 (2‐way ANOVA analysis).

Furthermore, to investigate whether other cell types behave similar to HNSCC cells in different bioinks, we bioprinted the breast cancer line MCF‐7 cells in TEMPO‐NC, Carboxy‐NC, and GelMAA bioinks and analyzed metabolic activity/ viability. Here, the luminescence signal was 70.2% lower in 3D than in 2D (**Figure** **5**b). This suggests that MCF‐7 suffer more from the bioprinting process than the HNSCC in regard to shear stress.^[^ ^24^, ^25^ ^]^ Comparing the three bioinks over the 21‐day culture period, the 3D bioprinted MCF‐7 bioconstructs showed a similar behavior regarding metabolic activity/viability as the HNSCC cells (Figure 5a,c). After bioprinting, MCF‐7 cells in TEMPO‐NC and Carboxy‐NC bioinks showed reduced viability on D03 and D06, appeared to recover on D12, and then showed a steep decline in TEMPO‐NC and a gradual decline in Carboxy‐NC until D21. In contrast in GelMAA, from D03 on metabolic activity/viability showed a steady rise. GelMAA supported metabolic activity to a higher degree, followed by Carboxy‐NC and TEMPO‐NC, similar to what was observed for the three HNSCC lines.

The viable cell number in MCF‐7 bioconstructs were twice as high as in HNSCC bioconstructs (Figure 5c) in all three bioinks. Most likely, this drastic difference observed is due to the higher proliferation rate of MCF‐7 cells. MCF‐7 cells have been shown to be robust and thus well exploited for 3D bioprinting.^[^ ^6^, ^30^, ^31^, ^32^ ^]^ Surprisingly, although HNSCC cells are rather sensitive in 2D culture, 3D bioprinting did not compromise their viability as much as that of MCF‐7 cells.^[^ ^1^, ^6^, ^30^, ^31^, ^32^ ^]^

Interpreting our results, we conclude that HNSCC cells derived from oropharynx and larynx as well as metastases behave differently in different bioinks.^[^ ^33^ ^]^ TEMPO‐NC was less compatible with HNSCC cells than Carboxy‐NC in favoring cell growth and proliferation. This could be due to the differences in the physiochemical properties of the two NC‐hydrogels. TEMPO‐NC has carboxyl (‐COOH) group whereas Carboxy ‐NC possess carboxymethyl group (‐CH_2_COOH) as their chemical structural backbones. Structurally in conjugation with alginate, TEMPO‐NC possess a heavier functional group (TEMPO‐group) than the Carboxy‐NC, thus possibly restricting hydrogel surface modification. At a biological/ cellular level, this could eventually hamper cell proliferation and migration. Whereas, GeMAA has its amine group in conjugation with alginate groups which gives mechanical freedom thus promoting cell migration. In conclusion, we propose Carboxy‐NC as novel bioink for TME bioprinting given that it behaves similar to GelMAA bioink but has various advantages as stated in **Table** **2**.

**Table 2:** Summarized differences between the *TEMPO‐NC*, *Carboxy‐NC*, and *GelMAA* hydrogels used in this study.

Properties	TEMPO‐NC *(TEMPO‐mediated oxidized tunicate NC)* ^[^ ^19^, ^47^, ^48^ ^]^	Carboxy‐NC (*Carboxymethylated tunicate NC)* ^[^ ^19^, ^47^, ^48^ ^]^	GelMAA *(Gelatin Methacrylate in sodium alginate)* ^[^ ^38^ ^]^
**Functional groups**	Hydroxyl groups of cellulose backbone partially substituted with **carboxyl** groups (COOH) mixed with alginate.	Hydroxyl groups of cellulose backbone partially substituted with **carboxymethyl** groups (CH_2_COOH) mixed with alginate.	Amine groups partially modified with **methacrylate** groups in alginate.
**Zeta potential**	−40.3≈– 57.2 mV (negatively charged nanofibrils)^a)^	−34.8 ± 2.9 mV (negatively charged nanofibrils)^a)^	Not applicable
**Endotoxin values**	≤0.5 EU m^−1^	≤0.5 EU m^−1^	≤50 EU m^−1^
**Surface area**	High	High	Depends on surface modified polymer
**Viscosity**	High	High	Low
**Printability**	High (even at 0.5 mL bioink used)	High (even at 0.5 mL bioink used)	Low or poor (<1 mL bioink used)
**Shape fidelity**	Favors for long term cultures	Favors for long term cultures	Varies based on cell conc. and bioink formulation
**Properties**	Good drug‐loading capacity, biocompatibility, & biodegradability and highly stable	Good drug‐loading capacity, biocompatibility, & biodegradability and highly stable	Improvable by further surface modification to enhance drug‐loading capacity, biocompatibility, biodegradability & stability
**Application**	In‐vivo drug delivery, disease modelling, wound healing, tissue engineering	In‐vivo drug delivery, disease modelling, wound healing, tissue engineering	Disease modelling, wound healing, tissue engineering

^a)^ The data on zeta‐ potential, and endotoxin of the NC‐hydrogels were obtained from the product specification listed on the website of Ocean TUNICELL, Norway. The link to the description is mentioned in the appendix.

### 2.4 Hydrogel Microstructures Reveal Differences in the Pore Size Correlated to the Varied HNSCC Cell Distribution in Different Hydrogels

**Figure 6:**
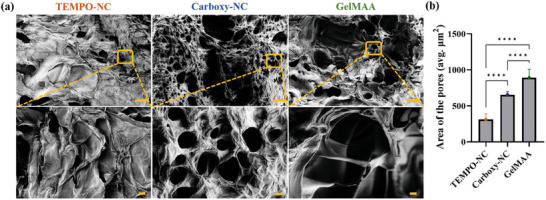
Microstructures of TEMPO‐NC, Carboxy‐NC, and GelMAA hydrogels. Microstructures of hydrogels crosslinked with 20 mm CaCl_2_ were visualized by SEM. (Scale: 100 µm for the images on the top and 20 µm for the images on the bottom). b) Average area of pores in three different hydrogels were analyzed by measuring the average area via ImageJ. (^****^ *p* < 0.0001).

Carboxy‐NC bioink constructs favoured survival of both HNSCC and breast cancer cells significantly better than TEMPO‐NC bioink constructs, similar to GelMAA. We hypothesize that this relates to differences in the hydrogel microstructure. Hence, we visualized TEMPO‐NC, Carboxy‐NC, and GelMAA hydrogels crosslinked with 20 mm CaCl_2_ by scanning electron microscopy (SEM) (**Figure** **6**a; Figure S2, Supporting Information). Both Carboxy‐NC and GelMAA showed an open pore structure with smooth surface, whereas the TEMPO‐NC showed rather a flat and rough surface characterized with closed pores. The closed pores observed in TEMPO‐NC hydrogel were extremely narrow, which could limit cell migration, thus favoring cell cluster formation. The relatively higher surface roughness may hinder cell growth, proliferation, and migration and may thus explain the lower increase in cell viability in TEMPO‐NC.^[^ ^34^ ^]^ Further, the average pore area differed significantly with 310, 652.7, and 890 µm for TEMPO‐NC, Carboxy‐NC and GelMAA hydrogels, respectively (Figure 6b). This fits well to the observed viability results. Of course, the freeze‐drying of the samples will to a certain extent generate a bias on the observed pore structure. However, we assume that all investigated hydrogels are affected by the freeze‐drying procedure in a similar way, so that we can avoid more complex fixation procedures, such as with tannic acid and osmium tetroxide.^[^ ^35^ ^]^ Additionally, it has been well documented that the pore‐size and the pore distribution significantly influences the cell positioning and migration.^[^ ^36^, ^37^ ^]^

We aimed to have a hydrogel with well‐distributed pores and a stable construct over the culture period that allows precise cell distribution. Based on bioconstruct handling, GelMAA tended to shrink after two weeks and appeared to degrade over time at the edges. This fits to literature data that report uncontrollable changes in long‐term stability of GelMAA structures.^[^ ^18^, ^19^, ^38^ ^]^ With the ultimate aim to achieve multicellular models, we envision to use cell‐specific bioinks full‐filling the specific needs of the cells of TME‐such as fibroblast, endothelial cells, and immune cells.

### 2.5 HNSCC Cells Proliferate in all Bioinks, Expressing Epithelial Phenotypes While Exhibiting a Distinct Cell Distribution

**Figure 7:**
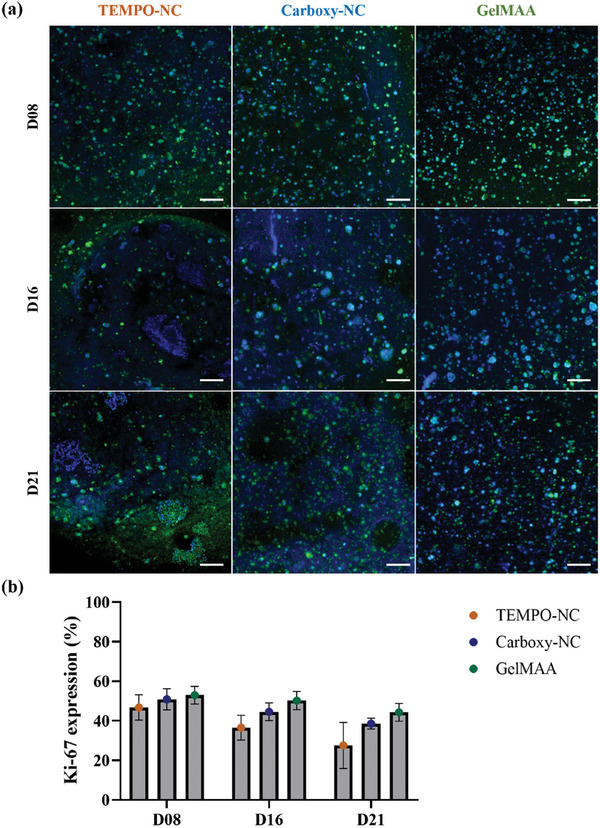
UM‐SCC‐22B proliferate and form defined‐ similar sized clusters predominantly in Carboxy‐NC and GelMAA. The bioconstructs were fixed and immunofluorescence (IF) staining was performed against‐ Ki67 (proliferation marker) and confocal imaging was performed a). The percentage of proliferating cells in different bioink constructs were calculated from the obtained confocal images b). Scale: 100 µm. Exemplary images from *n* = 3 experiments.

Our data indicates that HNSCC proliferated in the bioconstructs. However, the cell viability was lower than expected. To assess this in more detail, Ki‐67 (a proliferation marker) staining was performed in UM‐SCC‐22B bioprinted TEMPO‐NC, Carboxy‐NC, and GelMAA bioconstructs on D08, D16, and D21. UM‐SCC‐22B cells in all bioink constructs were positive for Ki‐67 indicating proliferation of 46.75%, 50.91%, and 52.94% of UM‐SCC‐22B cells on D08 in TEMPO‐NC, Carboxy‐NC, and GelMAA bioink constructs respectively (**Figure** **7**).

The rate of proliferation decreased from D08 to D16, however there was a higher drop from D16 to D21. This trend was prominent and significant for cells in TEMPO‐NC bioink constructs with a 41.02% drop in proliferating cells on D21. Both in Carboxy‐NC and GelMAA bioink constructs, proliferation decreased by 24.23% and 16.34% on D21. In comparison to the viability data obtained, the proliferation results were considerably diverse and difficult to compare directly. However, this discrepancy could be also due to the regions of interest (ROIs) chosen for visualization in specific areas of the construct. For instance, we stained three samples per condition choosing three ROIs (center, intermediate and outer regions representing eventual necrotic, quiescent and proliferative zones) of 200 µm z‐stacks per sample.^[^ ^7^ ^]^

**Figure 8:**
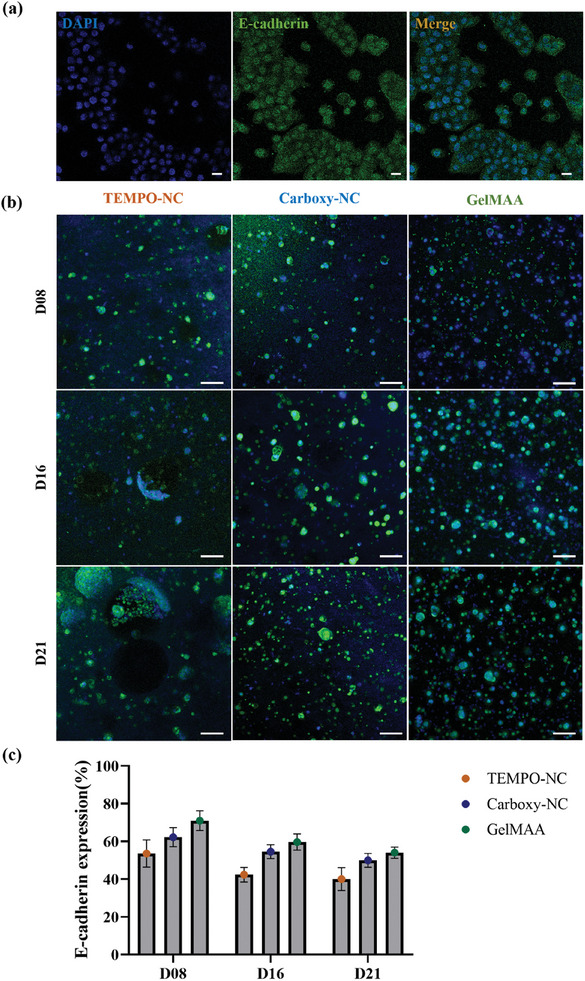
UM‐SCC‐22B proliferate and form defined‐ similar sized clusters predominantly in Carboxy‐NC and GelMAA. UM‐SCC‐22B cells in both 2D a) (Scale: 50 µm) and 3D bioprinted cultures b) (Scale: 100 µm) were stained for E‐cadherin‐ an epithelial biomarker. Initially, the 2D and 3D bioprinted bioconstruct samples were fixed, stained with immunofluorescence (IF) marker against E‐cadherin and imaged using confocal microscopy. The percentage of cells expressing their characteristic biomarker in different bioink constructs were calculated from the obtained confocal images c). Exemplary images from *n* = 3 experiments.

Most critical in these kinds of models is that the HNSCC cells maintain their epithelial phenotype and do not undergo epithelial‐mesenchymal transition (EMT).^[^ ^39^ ^]^ Thus, we stained for E‐cadherin (E‐cad, a characteristic epithelial marker) on days 08, 16, and 21. The majority of cells maintained E‐cad expression in 3D and in 2D culture (**Figure** **8**). We used the z‐stack images to calculate the percentage of cells that expressed E‐cad in the 3D constructs. Whereas, in 2D the majority of cells expressed E‐cad, the percentage of UM‐SCC‐22B cells expressing E‐cad in TEMPO‐NC, Carboxy‐NC, and GelMAA bioink on D08 were 53.55%, 62.24%, and 70.98% respectively. Here it appeared to have a large difference between TEMPO‐NC and GelMAA bioconstructs. Additionally, the duration of culture seemed to lower E‐cadherin expression. We are aware that there appears to be a discrepancy between 2D and 3D results. But deeper analysis would require to account for the limitation that certain information of cells in different z‐stack slices is lost, thus more sophisticated 3D imaging and analysis is required. On the other hand, this difference could indicate the onset of epithelial‐mesenchymal transition (EMT), during which cells progressively lose their epithelial characteristics and acquire mesenchymal properties. To address this, more‐detailed investigation for EMT biomarkers is necessary. However, staining performed in 3D and 2D cultures indicated co‐expression of the classical EMT biomarker ‐vimentin and E‐cadherin (Figure 8; Figure S3, Supporting Information). Recent studies have identified that in fact HNSCC cells can undergo partial EMT, resulting in a metastable state where cells express both epithelial and mesenchymal markers.^[^ ^40^ ^]^

The immunofluorescence (IF) images of Ki‐67 and E‐cad expression revealed that the cell distribution differed in all bioinks. This was most prominent in TEMPO‐NC where the cells formed large clusters, resembling cell monolayers on D16 and D21. Further, there were a lot of cell free zones visible. In contrast, in Carboxy‐NC and GelMAA, cells were well‐dispersed throughout the bioink. Clustering indicative of clonal cell proliferation was seen on D16 and D21. Cell clusters in both bioinks were similar in size with ≈3–8 cells. These observations befit the hydrogel microstructures seen in SEM micrographs. Especially in TEMPO‐NC, we observed large rough surfaces with closed pores. This appears to rather reflect the 2D culture surface and supports the observation of cell growth as monolayer rather than in 3D.

We provide detailed evidence data that HNSCC cells can be bioprinted in tunicate‐NC. Tunicate Carboxy‐NC similar to the synthetic GelMAA bioink maintain their proliferative and epithelial phenotype over a prolonged culture of 21 days. The hydrogel microstructure fits well to the dispersion and growth pattern of the cells in the hydrogels. Importantly, all three tested HNSCC cell lines appeared to survive the bioprinting process similar to the compared breast cancer cell line MCF‐7, often used in bioprinting approaches. Cell‐type specific differences were observed which eventually correspond to their tissue source and tumorigenic nature such as being metastatic. By adding other cell types to build the TME, further refinement may be required: for instance, improving the culture medium cocktail, improving surface functionalization, modifying of NC‐to‐alginate ratio (altering other bioprinting parameter accordingly).^[^ ^22^, ^24^, ^25^, ^27^, ^29^ ^]^ Ideally, one biomaterial that fits all cell types and requirements can be established.

For HNSCC, to the best of our knowledge, only one group achieved a bioprinted HNSCC in vitro tumor model so far, published 2021 and 2023.^[^ ^7^, ^41^ ^]^ They introduced a HNSCC bioprinted model using a porcine tongue‐derived decellularized extracellular matrix composite containing alginate and gelatin. In that study, UM‐SCC‐12 and −38 cells were used and printed also with an extrusion bioprinter with 1 × 10^6^ cells mL^−1^ to a disc with a 5 mm diameter and 500 µm height. Samples were cross‐linked with 100 mm CaCl_2_. Their bioink showed a rather fibrous network with nanometer‐sized pores contrary to the NC‐based hydrogel we present in this study. Within this fibrous structure, cells showed a high cell viability and spheroid‐like growth, most likely related to the growth factors present within the decellularized ECM. Although decellularized matrix extraction is a notoriously laborious process, it also introduces unknown factors that could lead to contamination, potentially causing an inflammatory response.^[^ ^9^, ^10^, ^14^ ^]^ The unknown factors present in decellularized matrices could potentially trigger complex immune responses upon recellularization, leading to either overridden or amplified cell‐specific immune signals.^[^ ^42^ ^]^ To address such biomaterial induced responses, the decellularized biomaterial has to be tailored according to the tissue/ cell type of interest.^[^ ^9^, ^10^, ^14^ ^]^ On the other hand, as indicated in Table 2, the use of GelMA has its own challenges such as temperature instability, shear thinning, cell‐specific surface functionalization and maintenance of shape fidelity. Conversely, NC provides a neutral biomaterial that is stable, highly tunable and offers a platform to customize its properties according to the cell type used with minimum modifications. For example, arginine–glycine–aspartic acid (RGD) functionalization can be employed when bioprinting fibroblasts to stimulate cell adhesion, while vascular endothelial growth factor receptor (VEGFR) functionalization can be used when bioprinting endothelial cells to regulate angiogenesis similar to recapitulate in vivo conditions. Therefore, this work lays the groundwork for developing a 3D bioprinted model for HNSCCs utilizing a novel and innovative NC‐based bioink.

### 2.6 Functional Validation Proves Efficacy of Treatment Modalities in 3D Bioprinted Models‐ Both Cell Line and Patient‐Derived HNSCC Cells

**Figure 9:**
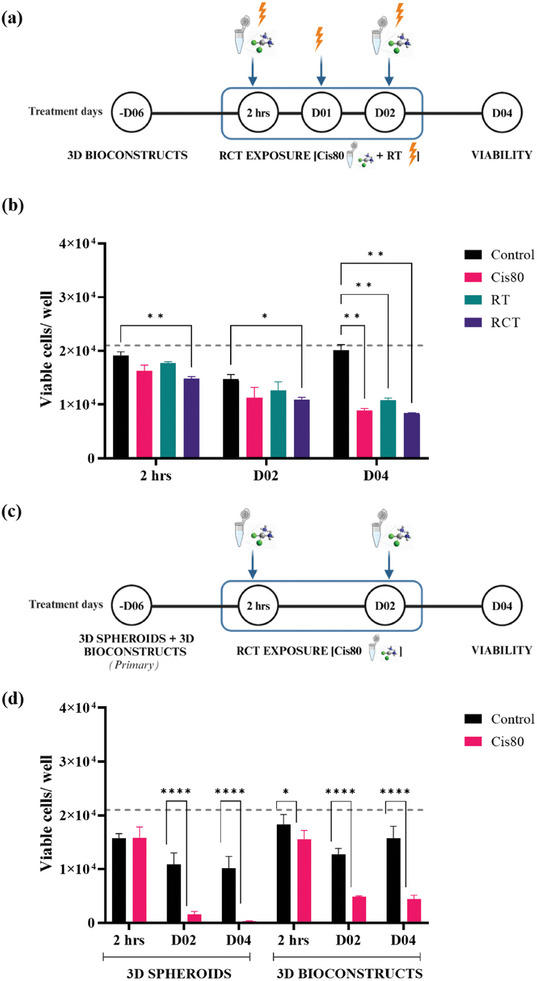
Functional validation proves efficacy of treatment modalities in 3D bioprinted models‐ both cell line and patient‐derived HNSCC cells. a) UM‐SCC‐22B cell‐laden Carboxy‐NC bioconstructs were exposed to RCT on days 00–02. b) The viability was measured at the indicated time points documenting the cytotoxic effect of RCT on the bioconstructs. c) Patient‐derived HNSCC cells were fabricated into both 3D spheroids and 3D bioconstructs and exposed to Cis80. d) The measured viability indicated a stronger effect on the spheroids than in the bioconstructs on D02 and 04. The line indicates the initial bioprinted cell number (= 21 000 cells/ / 4.2 µL bioink). N‐3 independent experiments; *n* = 3 samples per condition for b) and N‐3 different patient‐derived samples; *n* = 5 samples per condition for d) ^*^ *p* < 0.05, ^**^ *p* < 0.005, ^***^ *p* < 0.0005, ^****^ *p* < 0.0001 (2‐way ANOVA analysis).

After optimizing our 3D bioprinted model, we progressed to investigate the model's functional response. Clinically, advanced HNSCCs are often treated with a combination of radiotherapy and sequential chemotherapy. We adopted established radiochemotherapy (RCT) protocol to our 3D bioprinted constructs.^[^ ^21^ ^]^ Precisely, the bioconstructs were exposed to fractionated irradiation (RT) over three days and consecutive chemotherapy on two days with the platinum‐based chemotherapeutic agent, cisplatin, at a concentration of 80 µm (Cis80) (**Figure** **9**a,b).

Already 2 h after Cis80 treatment, we observed a 15.15% reduction in cell viability, which progressed to 55.78% on D04. RT alone led to a slight 7.57% decrease, which progressed on D04 to 46.12%. While combined RCT led to a slightly higher 22.71% decline, which continued to an overall 58.33% decrease.

While RT induced rapid cell death in HNSCC cells in 2D, 3D spheroids and explant models,^[^ ^43^ ^]^ it exerted delayed efficacy in 3D bioprinted models. This may result from the limited radiation penetration and the radioprotective properties of the hydrogel matrix.^[^ ^43^ ^]^

As a final proof, we upgraded the model with patient‐derived HNSCC cells derived from three independent donors representing distinct anatomical sites of origin‐ oral cavity, oropharynx, and larynx and exposed them to Cis80 (Figure 9c,d). As a comparative system, a well‐established in‐house 3D primary HNSCC spheroid model was fabricated.

Importantly, all three patient‐derived cells showed a similar growth behavior in 3D bioprinted constructs over the 10 days like the cell lines (Figures 4 and 5). This proves bioprintability of patient‐derived HNSCCs.

Although the same cell numbers were seeded, only 50% of the cells were incorporated into the spheroids (Figure 9d; Figure S4, Supporting Information). Three days after initiated‐culture, the spheroids and bioconstructs accounted for a viable cell number‐ 9836 and 19186 cells per sample respectively. This highlights the advantage of 3D bioprinting that all cells can be embedded within the hydrogel matrix. Further, seeding the cells into spheroids or culture of patient‐derived organoids will select for certain cell sub‐types. This has been described for patient‐derived organoids that they specifically favor stem‐cell like sub‐types.^[^ ^44^ ^]^

Regarding patient‐derived cells, the 3D bioprinted model revealed a Cis80 effect already 2 h after treatment, while in the 3D spheroid model it had no effect at this early time point. From D02 and D04 the viability dropped approximately ≈85.77% and 90.41% in the spheroid system while 61.68% and 71.99% in the bioprinted model, in comparison to their respective controls. Given that the drug efficacy in patients is not 100%, herein 3D bioprinted models appeared to better reflect this in vivo situation than the spheroids which may overestimate the drug toxicity.^[^ ^4^, ^5^ ^]^

## 3 Conclusion

The HNSCC cells showed nearly equivalent viability in Carboxy‐NC bioink similar to the frequently used GelMAA and better survival than in TEMPO‐NC over a long‐term culture period of 21 days. The three different HNSCC cell lines showed differing metabolic activities that may relate to the tissue origin and grade of the tumor, the cell lines have been generated from. The Carboxy‐NC bioink promoted quite similar HNSCC bioconstruct microstructure as GelMAA favoring comparable cell distribution and proliferation. Due to its numerous advantages, most importantly high biocompatibility and low immunogenicity, we regard it as excellent bioink to proceed with further studies aiming at high‐throughput pre‐clinical drug testing studies for HSNCCs.^[^ ^10^, ^12^, ^13^, ^19^ ^]^ Importantly, the bioconstructs allow for the treatment modality testing, not only for the cell lines but also for patient‐derived HNSCC cells. Compared to the spheroid models, the cytotoxic effects were more reflecting the response in patients.

After having demonstrated the proof‐of‐concept, next steps to advance the current model to heterotypic ones are to optimize the bioprinting process for other cell types (fibroblasts, endothelial, and immune cells) in the TME, individually, through a similar re‐iterative process. Such a heterotypic model could be applied to study drug effectiveness, toxicity, drug resistance, and the complex mechanisms behind these phenomena in a preclinical setting.

## 4 Experimental Section

### Cell Source

The HNSCC cell lines used were UM‐SCC‐14C, UM‐SCC‐11B, and UM‐SCC‐22B (derived from different tumor sites, oral cavity, larynx, and hypopharynx, respectively, the University of Michigan, USA).^[^ ^21^, ^26^ ^]^ HNSCC cells were maintained in culture media containing Eagle's minimum essential medium (EMEM, Carl Roth GmbH + Co. KG, Germany) supplemented with 10% fetal bovine serum (FBS, Gibco, Thermo Fisher, USA) and 1% antibiotic‐antimycotic mix (Antibiotic‐Antimycotic, Gibco, Thermo Fisher, USA). The breast cancer cell line‐ MCF‐7, derived from a 69‐year‐old Caucasian metastatic breast cancer (adenocarcinoma, Michigan Cancer Foundation, USA) was additionally used. MCF‐7 cells were maintained in Dulbecco's Modified Eagle Medium (DMEM) (Thermo Fischer, USA) supplemented with 10% FBS and 1% antibiotic‐antimycotic mix (Antibiotic‐Antimycotic, Gibco, Thermo Fisher, USA). All the cells used in this study were cultured in T‐75 flasks (Greiner, Sigma‐Aldrich, USA) retaining conventional cell culture conditions in an incubator (humidified environment at 37 °C with 5% CO_2_) with a growth media change every 3 consecutive days. Upon confluence (85%–90% confluence), the cells were detached with 2% trypsin (Sigma Aldrich, USA) and counted manually using a haemocytometer. For the continuing culture, the cells were passaged with a seeding concentration of 3 × 10^5^ cells/ T‐75 flask.

For the functional assessment of the 3D bioprinted model, primary HNSCC cells were used. These were obtained from three donors, after having obtained informed consent in accordance with the Declaration of Helsinki (Ethical Approval No. 2018–603N‐MA, the Ethics Committee of the Medical Faculty of Mannheim, University of Heidelberg,). On the day of surgery, parts of the resected tumor tissues were collected. After mincing into fragments ≈2 mm^2^ in size, these were incubated in Liberase DH solution (1:100 dilution in Dulbecco's PBS with calcium and magnesium; Roche, Germany; Gibco, Thermo Fisher Scientific, USA) at 37 °C on a shaker for 30 min to isolate the epithelial tumor cells. The tissue suspension was filtered through 100 µm strainers and centrifuged. The resulting cell pellet was cultured in T‐75 flasks using PneumaCult‐Ex Plus basal medium supplemented with PneumaCult Ex Plus Supplement (Stemcell Technologies, Canada) according to the manufacturer's instructions, 1% L‐glutamine (Carl Roth GmbH + Co. KG, Germany), 1% antibiotic‐antimycotic mix (Antibiotic‐Antimycotic, Gibco, Thermo Fisher Scientific, USA), and hydrocortisone (96 µg mL^−1^; Stemcell Technologies, Canada).^[^ ^45^ ^]^ The cultures were maintained at 37 °C in a humidified atmosphere with 5% CO₂, and cells were harvested and/ or passaged when they reached ≈80% confluency. Upon harvest, to establish 3D bioprinted‐ 5 × 10⁶ cells mL^−1^ bioink and for 3D spheroid models‐ 21000 cells well^−1^ were used. The bioprinted models were cultured in 96‐well flat‐bottom plates, while spheroids were maintained in Nucleon Sphera 96‐well ultra‐low attachment (ULA) plates (Thermo Fisher Scientific, USA). Both models were cultured for 10 days under identical conditions before further analysis.

### 3D Bioprinting—3D Model Design

The primary step in fabricating a 3D bioprinted model is to design a reproducible 3D structure that correlates to the goals of the study. First, the bioprinting parameters were defined and optimized such as‐ cell density, growth media volume, growth factor concentration, print head and print‐bed temperature, printing speed & pressure, number of layers, printing pattern, infill density, nozzle/ needle size, crosslinking method and tailored post‐print sample characterization protocols according to the system.^[^ ^22^, ^24^, ^27^, ^46^ ^]^ Herein, the bioprinting parameters were optimized to achieve a well highly printable, cell‐friendly model that promotes cell growth, proliferation, and migration (step‐by‐step process illustrated in Figure 9).^[^ ^46^ ^]^ A simple 3D cylinder with dimensions 3 × 0.6 mm (≈4.2 µL volume) was designed using Fusion 360 software (Autodesk INC, USA). The designed 3D structure (in.stl format) was exported as a gcode format, which was readable by the bioprinter (BIOX CELLINK, Sweden). The optimized bioprinting parameters are given in Table 1.

### 3D Bioprinting—Bioink Preparation

In this study, two different tunicate‐derived NC‐based bioinks, TEMPO‐NC: TEMPO‐mediated oxidized NC and Carboxy‐NC: carboxymethylated NC (Ocean TUNICELL, Norway) were tested compared to the gelatin‐based bioink, GelMAA (GelMA A, CELLINK AB, Sweden) (Table 2). The NC hydrogels were mixed with 3% w/v of alginate (purchased from Pronova SLG 100 Ultrapure, sodium alginate, Novamatrix, IFF Nutrition Norge AS, Norway) in 4.6% w/v of D‐mannitol (Sigma Aldrich,) at a ratio of 2:1, using luer‐lock syringes. Both syringes were connected using luer‐lock connectors and the contents were mixed up to 200 times avoiding air‐bubbles. GelMAA, was prepared according to the manufacturer's protocol.

Then, a prepared mixture of 1 mL of NC in alginate and GelMAA hydrogel were mixed with the HNSCC cells re‐suspended in 200 µL of media/1 mL bioink. 1 × 10^5^, 1 × 10^6^, 1 × 10^7^ cells mL^−1^ of bioink were tested. The cell‐hydrogel mixture was mixed slowly using luer‐lock syringes. Once the bioink was prepared, 1 mL of bioink was loaded into the bioprinting cartridges (CELLINK AB, Sweden) using luer‐lock connectors (CELLINK AB) and set to be bioprinted.

### 3D Bioprinting—Bioprinting

Once the bioink was prepared, the bioprinter was sterilized and calibrated with cartridges loaded with their respective bioink. After bioprinter calibration, the cell‐laden bioink was bioprinted in the form of cylindrical constructs into 96‐well plates (Corning, USA) (see Table 1). Depending upon the experimental condition at least 3–48 bioprinted constructs were then chemically crosslinked with 100 µL of CaCl_2_ (CELLINK, Sweden) for 5 min. 50 and 20 mm CaCl_2_ were tested. After 5 min, the crosslinker was aspirated and 200 µL of the growth media cocktail added. 3D bioprinted constructs were maintained for 21 days in sterile conditions in an incubator and 50% of the media was changed every third day.

### Radiochemotherapy (RCT) to 3D Bioprinted HNSCC Constructs

To investigate the functionality of the 3D bioprinted HNSCC model (UM‐SCC‐22B), the bioprinted constructs were subjected to radiochemotherapy (RCT) treatment. The bioconstructs were cultured over a period of 10 days. Three days after bioprinting, their mean viable cell number per construct was calculated using the ATP assay as described in section 4.5.1. Six days later after bioprinting, the bioconstructs were treated with platinum‐based chemotherapy drug, cisplatin (Selleckchem, USA) at a concentration of 80 µm (Cis 80) on days 00 and 02. In addition, they were exposed to fractionated irradiation (RT) on days 00, 01, and 02 at a dose of 2 Gy with 2 cm of polymethylmethacrylate slabs placed above and 5 cm below the 96‐well plate to simulate clinical irradiation conditions. RT was delivered using a medical linear accelerator (Synergy; Elekta AB, Stockholm, Sweden) with a photon energy of 6 MV. The RCT protocol was tailored based on the standard treatment regimen for HNSCC patients and from the preliminary data from the established in‐house 2D and 3D spheroid HNSCC models.^[^ ^21^, ^49^ ^]^ 2 h later (2 h), two days (D02) and four days (D04) after initial treatment, their viable cell number was assessed (Figure 9a).

### Chemotherapy Treatment to Patient‐Derived 3D Bioprinted and 3D Spheroid Models

The patient‐derived 3D bioprinted HNSCC constructs and spheroids were exposed to platinum‐ based chemotherapy drug, cisplatin at a concentration of 80 µm as described in section 4.3 (Figure 9c). As for viability testing, the spheroids have to be transferred from ULA plates to white‐bottomed chemiluminescence‐based viability measurement plate, there is a high possibility that all the cells that were not incorporated in the spheroids were left behind and not counted.

### Characterization—Cell Viability Analysis

To evaluate the metabolic activity/viability of cells within the bioprinted constructs, an ATP based chemiluminescence cell‐viability quantitative assay kit was used according to the manufacturer's instructions (3D CellTiter‐Glo Promega, USA). Briefly, the bioconstructs were transferred to an opaque 96‐ well plate and washed with PBS thrice with 5 min resting time. After washing, 100 µL of the viability kit reagent was added and shaken for 5 min and then incubated for 30 min at room temperature (R.T.). Then, the luminescence signal was measured using a plate‐reader (Infinite 200 PRO, TECAN, Switzerland). The cells were monitored for their viability on days 00, 03, 06, 12, 14, 16, and 21.

### Characterization—Immunofluorescence Staining (IF)

To examine whether the cells in different bioink constructs maintain expression of their characteristic cell specific marker E‐cadherin‐ an epithelial marker, downregulated upon epithelial‐to‐mesenchymal transition (EMT), immunofluorescence staining (IF) was performed on days 08, 16, and 21. In addition, proliferation was measured by Ki‐67 staining. Ki‐67 is a nuclear protein expressed particularly during late G1, S, G2, and M phases in the cell cycle. The bioconstructs were fixed with 4% paraformaldehyde (PFA) overnight, permeabilized with IF wash buffer (containing PBS, 500 mg BSA (0.1% w/v), 1 mL Triton X‐100 (0.2% v/v), 0.25 mL TWEEN 20 (0.1% v/v)) and blocked with 5% normal goat serum (v/v) (BIOZOL Diagnostica Vertrieb GmbH, Germany) against E‐cadherin (1:50; Anti‐E Cadherin ab40772, abcam, UK or against Ki‐67 (1:20, Ki67, Biozol, Germany) to monitor cell proliferation overnight at 4–8 °C. After washing, the samples were stained with Alexa‐488 (1: 200 dilution, anti‐rabbit, Sigma–Aldrich). All constructs were counter stained with DAPI as a nuclear dye (Thermo Fischer). After 3 washing steps with IF buffer and PBS, the constructs were embedded in 2% agarose (Sigma–Aldrich) in a 35‐mm glass‐bottomed dish (µ‐Dish 35 mm, ibidi, Germany) with PBS. The samples were imaged using a confocal microscope (TCS SP8 upright laser scanning microscope, Leica, Germany) with a z‐stack depth of 200 µm. Each sample was imaged in three sections of 200 µm z‐stacks representing the top, middle, and bottom portions. Number of replicated used per condition was three (*n* = 3). The images were analyzed using Fiji software (Fiji is just ImageJ software).^[^ ^50^ ^]^ The z‐stack images were loaded into Fiji, and the channels were split after proper channel assignment. The split channels were converted into a single image using maximum projection, and then the channels were merged. The merged channel images were despeckled to remove artifacts. This image processing was performed iteratively, and the processed images were analyzed for biomarker expression. To determine the co‐expression of Ki‐67 or E‐cadherin expression, the processed images were loaded in ImageJ and image thresholding was performed for both DAPI (blue) and Ki‐67/E‐cadherin (green) channels. After thresholding and noise removal, both the channels were combined to assess co‐localization using the image calculator option. The arrived co‐localization image was processed by image thresholding and converted into a binary mask. The masks were then superimposed onto the composite merged image and the number of cells expressing both DAPI and Ki‐67/E‐cadherin were counted. The percentage of cells expressing respective biomarkers were calculated and graphed. All the images presented in this paper were from the mid‐section.

### Characterization—Scanning Electron Microscopy of the 3D Bioprinted Constructs

To examine the microstructure of the hydrogels used in this study, Scanning Electron Microscopy (SEM) was performed. The hydrogels were prepared as described without the addition of cells and casted onto a flat bottom 12 well plate (Corning) in the form of a cylinder and crosslinked chemically with 20 mm CaCl_2_ for 5 min. Then the crosslinker was aspirated and PBS added and incubated at 37 °C with 5% CO_2_ overnight. The hydrogel samples were, sliced using a scalpel and lyophilized (Alpha 3–4 LSC basic, Christ, Germany) with a cold trap temperature of −105 °C. Lyophilized dried samples were then mounted on to metallic stubs using carbon tape, and sputter coated with a thickness of 6 nm using an 80/20 mixture of gold/palladium (Leica Microsystems). Micrographs were procured using Zeiss Leo 1530 instrument at the desired magnification range and scans were performed at 2.0 kV accelerating voltage. ImageJ software was used to calculate the porosities of the hydrogel samples. The pore area and its dimensions were determined manually using a re‐iterative methodology.^[^ ^51^ ^]^ First, the pixel size was converted by thresholding procedure. This was followed by image segmentation, i.e., converting the threshold corrected image to a binary mask and morphological filtering was performed by correlating the obtained segmented mask to the original SEM image. The arrived mask after morphological filtering was verified by a re‐iterative process until the segmented mask approximately equated to the original SEM image pores. After the verification step, quantitative data such as pore size, area, and number of pores were extracted, and statistical analysis was performed.

### Statistical Analysis

Three independent experiments (n‐3) in triplicates each were performed. Acquired data were analyzed via two‐tailed unpaired t‐test or one‐way or two‐way ANOVA and plotted with mean ± standard deviation (SD) (GraphPad Prism 9.5.1.733, USA). The p values < 0.05 were considered significant and indicated as mentioned: ^*^ *p* < 0.05, ^**^ *p* < 0.005, ^***^ *p* < 0.0005, ^****^ *p* < 0.0001.

## Conflict of Interest

The authors declare no conflict of interest.

## Supporting information



Supporting Information

## Data Availability

The data that support the findings of this study are available on request from the corresponding author. The data are not publicly available due to privacy or ethical restrictions.
