# Supplementary material for: 3D Bioprinted Head and Neck Squamous Cell Carcinoma (HNSCC) Model Using Tunicate Derived Nanocellulose (NC) Bioink
Source: Adv Healthc Mater. 2025 Jan 13;14(7):2403114. doi: 10.1002/adhm.202403114 (PMC11912098; doi:10.1002/adhm.202403114)
Supplement: Supplementary file 1 — Supporting Information [file ADHM-14-0-s001.docx]

Supporting Information

**Table S1. Difference between different tumor models.** The difference between different tumor models- 2D in vitro, ex vivo/ explants, 3D spheroid, organoids and bioprinted models were distinguished based on their advantages and limitations.

| **Tumor model** | **Advantages** | **Disadvantages** |
| --- | --- | --- |
| **2D model** | - High throughput  - Low cost  - User-friendly with established culture and characterization protocols | - Limited relevance to native physiology  - High possibility for genetic drift or acquired mutations  - Minimal / no cell-matrix interactions |
| **Explant** | - High physiological relevance, retaining the native extracellular matrix  - Potential for automation  - More cost-effective than animal models  - High throughput possible (depending on tumor size; required cohort studies) | - Limited access to patient tumour tissue with equivalent pathophysiology  - High risk of contamination  - Experimental ease and reproducibility are constrained by sample availability and variability |
| **3D spheroids** | - Facilitates cell-cell and cell-matrix interactions  - High throughput and reproducibility  - Cost-effective  - User-friendly with established protocols | - Prolonged cultures can lead to cell aggregation  - Formation of heterogeneously sized spheroids  - Lack of native tissue-like spatial organization, restricting oxygen and nutrient transfer |
| **3D organoids** | - Resemble native tissue organization  - Scalable culture model  - Support long-term culture  - Enable biomarker identification  - Moderate cost | - High variability in the size and shape of organoids  - Limited throughput and reproducibility  - Labor- intensive and time- consuming culture protocols |
| **3D bioprinted constructs** | - Facilitate cell-cell, cell-matrix, cell-immune, cell-vascular network interactions  - Allow studies of multicellular effects  - High physiological relevance  - Relatively high level of automation  - High throughput and reproducibility  - Moderate cost | - Multicellular models require extended printing times  - Limited ability to geometrically mimic native tissue  - Challenges in balancing biocompatibility, printability and cell-specific growth needs  - Difficulty in selecting hydrogels compatible with multicellular systems and drug interactions |

**Information S1. Biochemical properties of the hydrogels**. Table 2 enlists the information on functional groups, their respective zeta potentials, surface area, viscosity, bioburden, endotoxin values including properties such as, drug-loading capacity, biocompatibility and biodegradability and their respective applications were extracted from the product description provided by the company, Ocean TUNICELL, Norway and CELLINK, Sweden. Link to the product description has been mentioned below:

- TTC hydrogel- <https://oceantunicell.com/wp-content/uploads/2021/08/MSDS-TTCM-version-2-170821.pdf>
- CTC hydrogel- <https://oceantunicell.com/wp-content/uploads/2021/08/MSDS-TTCM-version-2-170821.pdf>
- GelMAA hydrogel- <https://www.cellink.com/wpcontent/uploads/2022/03/GelMA-A-SDS-12-July-2019-4.pdf>

**Supplementary Figure 1: UM-SCC-11B cells in NC-bioink indicated vacuolization**

**
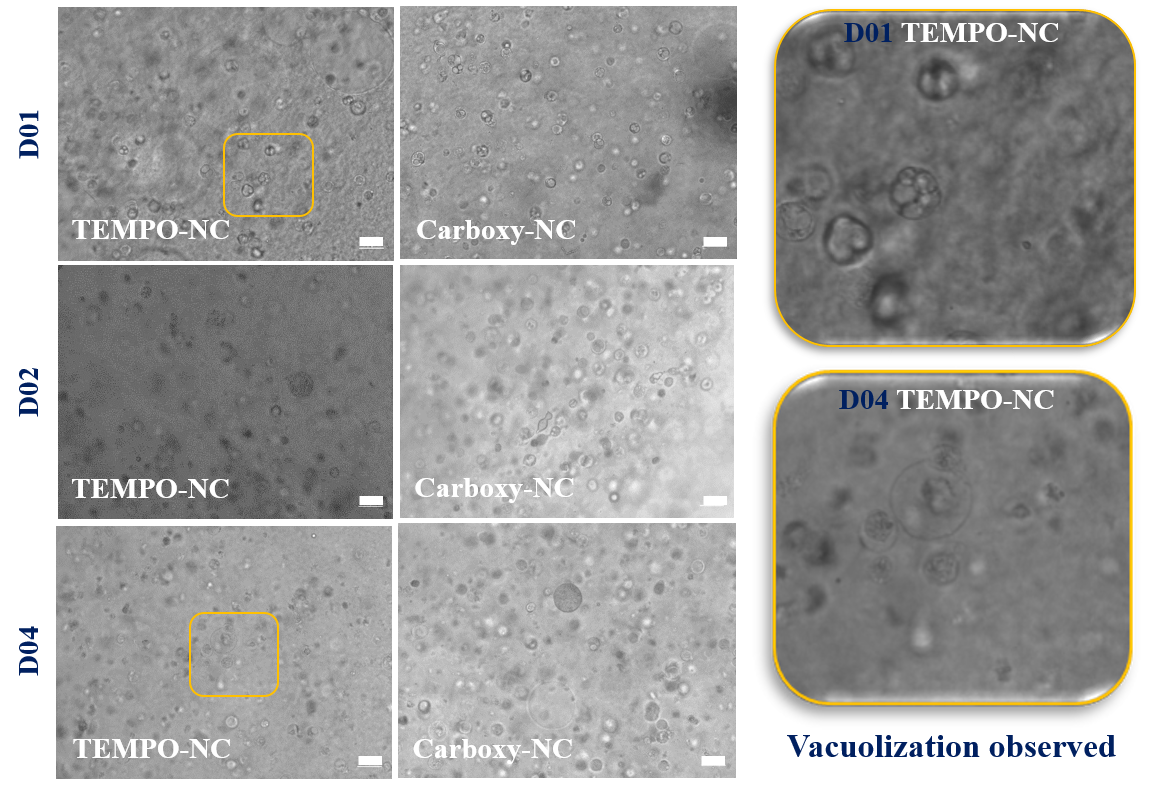
**

**Figure S1.** UM-SCC-11B cells in NC-bioink indicated vacuolization (D00-04). Vacuole formation was observed in different HNSCC bioprinted constructs in TEMPO-NC and Carboxy-NC bioinks. The vacuoles formed have been zoomed and visualized right side to the microscopic images. (Scale: 50 µm).

**Supplementary Figure 2:** **Hydrogel microstructures revealed difference in the pore size with open and closed pores**


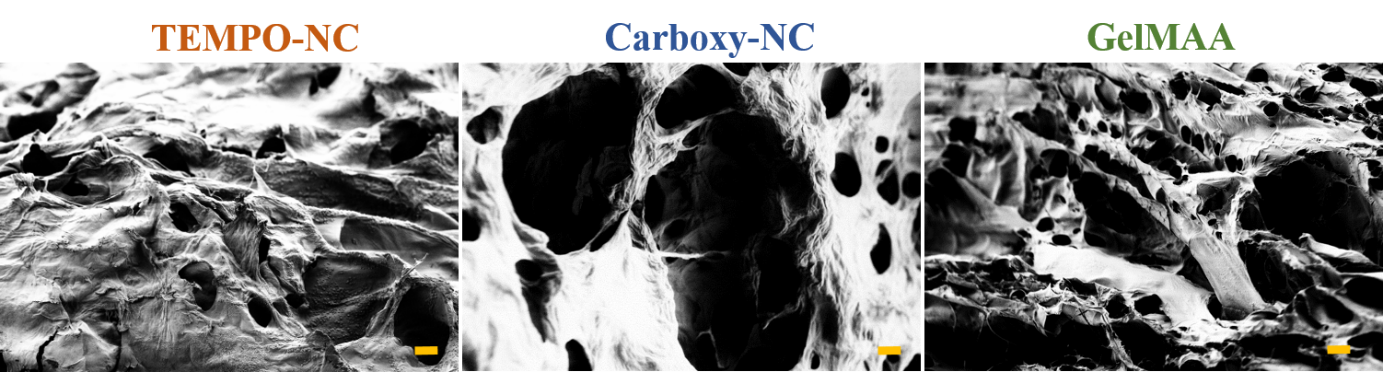


**Figure S2.** Hydrogel microstructures revealed difference in the pore size with open and closed pores. Microstructures of hydrogels crosslinked with 20 mm CaCl_2_ were visualized by SEM. (Scale: 20 μm).

**Supplementary Figure 3: UM-SCC-22B cells in Carboxy-NC bioink expressed EMT biomarker- vimentin in 3D and 2D cultures**


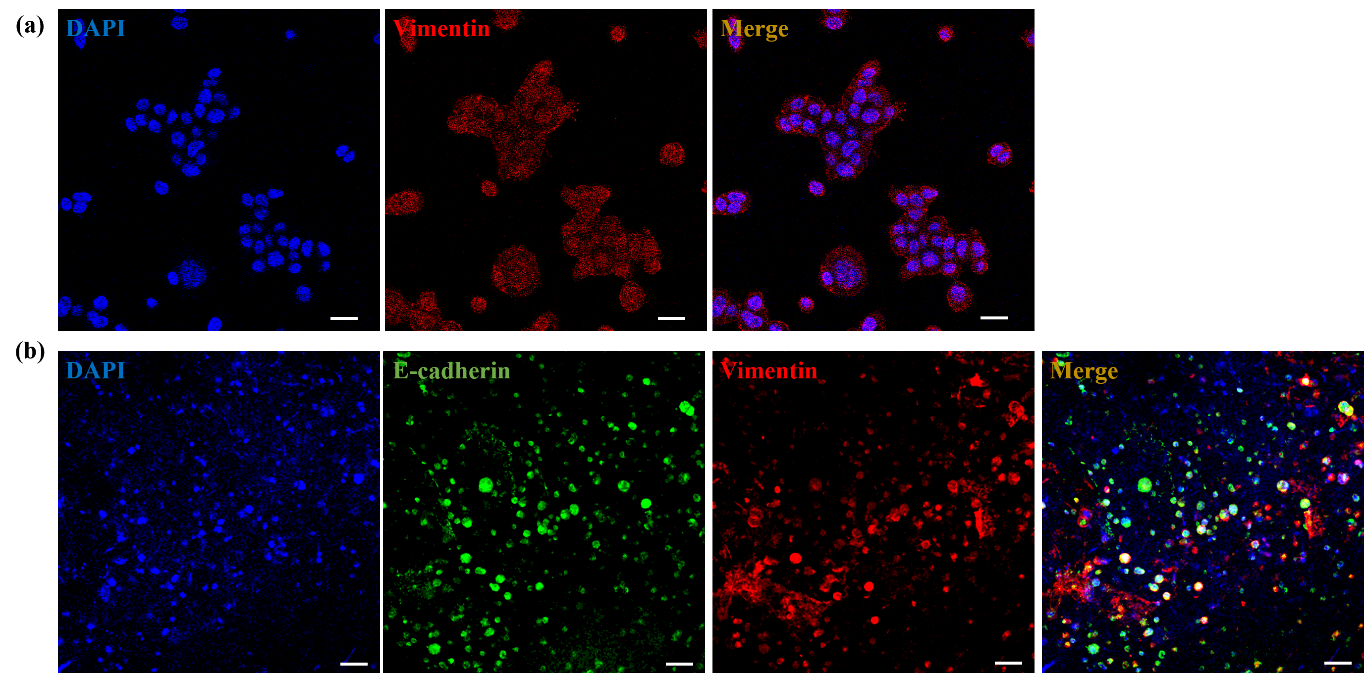


**Figure S3.** UM-SCC-22B cells in Carboxy-NC bioink expressed EMT biomarker- vimentin in 3D and 2D cultures in 2D (a) (Scale: 50 µm) and 3D (b) (Scale: 100 µm) cultures.

Supplementary Figure 4: Different patient-derived HNSCC cells with varied tissue origin formed different sized spheroids


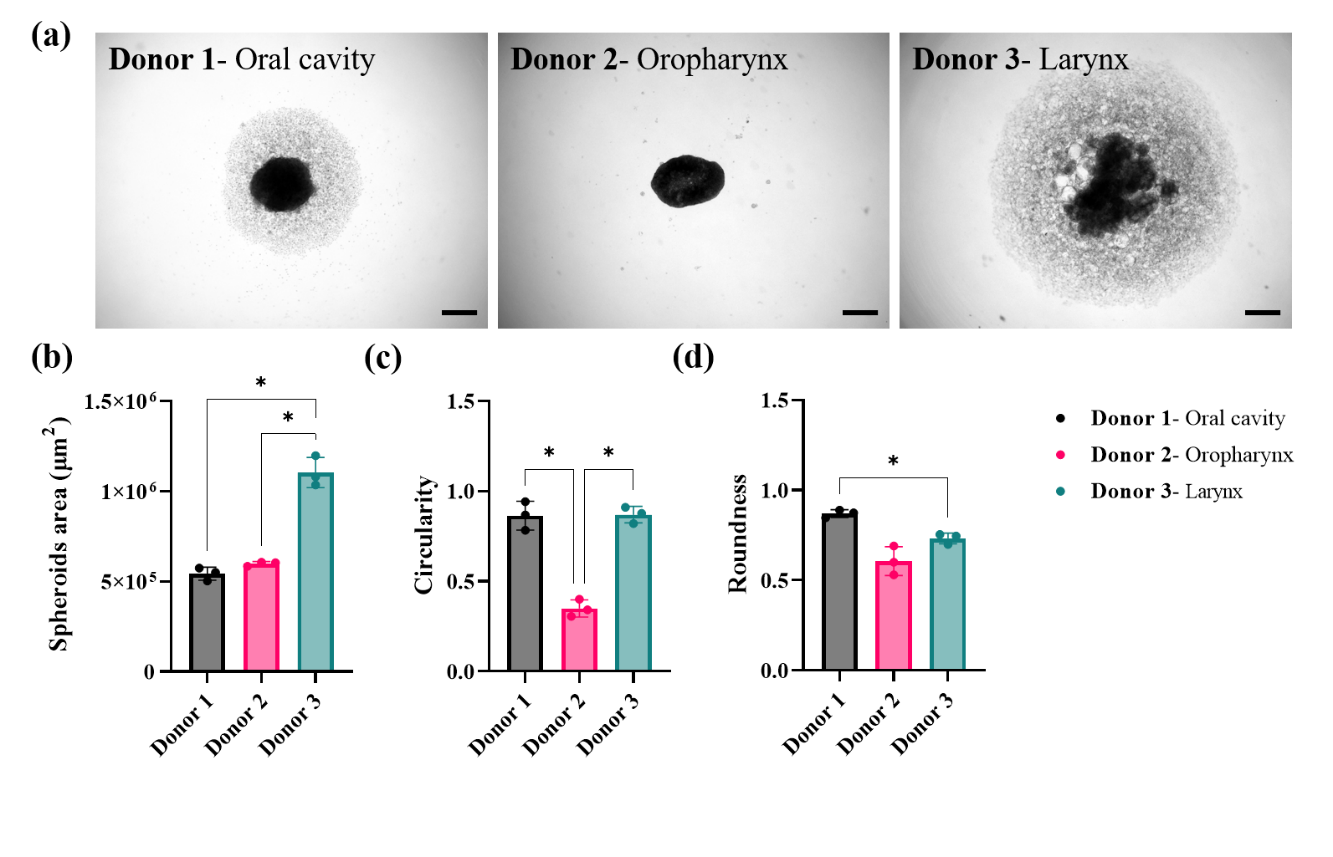


Figure S4. Different patient-derived HNSCC cells with varied tissue origin formed different sized spheroids. (a) Microscopic images of D03 spheroids generated from three different donor derived tissues. Quantitative analysis of the generated spheroids were investigated based on their (b) area, (c) circularity and (d) roundness. Donor 3-derived spheroids had a significantly larger surface area of 1104,609 µm^2^ compared to those from donors 1 and 2 with respective areas of 543,048 µm^2^ and 598,382 µm^2^ (Figure S4 (b)). Additionally, spheroids from donor 2 were observed to be elliptical with a circularity of 0.034 units, in contrast to the more rounded spheroid morphology observed in donor 1 and donor 3 with a their respective circularities of 0.8643 and 0.8697 ((c) and (d)). Such variations in spheroid morphology indicate significant differences in nutrient and oxygen transfer rates, which could impact the consistency and reproducibility of high-throughput screening (HTS) studies. (Scale: 100 µm)
